# Dr. James Edward Prichard

**Published:** 1901-03

**Authors:** 


### Obituary.

---

### Dr. JAMES EDWARD PRICHARD.

It is with sincere regret that we have to announce the death of Dr. James Edward Prichard, which took place at his house in Redland Road on February 2nd, 1901. More than a year ago Dr. Prichard suffered from symptoms of a growth in the rectum, but it was not until too late that he sought advice, and then the condition was found not to admit of any radical cure. He went on with his work until last July, when an inguinal colotomy was performed to give him temporary relief. He bore with great fortitude the pain and irremediable discomfort of the last few months of his life, and he died from exhaustion consequent upon the extension of disease in the abdomen.

Dr. Prichard was the second son of the late Augustin Prichard, and grandson of Dr. James Cowles Prichard, F.R.S., the ethnologist, and was born at the Red Lodge in Park Row in 1849. He first went to school at Mr. Smith's, in Richmond Terrace; but when Clifton College began, this school was swallowed up in it, as some others were at that time, and Dr. Prichard was one of the first College boys. From Clifton he went to Oxford in 1869, and obtained the Hody Exhibition at Wadham for Hebrew. Intended originally for the Church, Oxford altered his ideas, and after taking his B.A. degree with honours, he went to University College, London, to study medicine. He held the post of House Physician under the late Dr. Wilson Fox, and became a Member of the Royal College of Surgeons in 1878, proceeding to the M.B. degree at Oxford in 1879. After leaving London, he was for a short time House Surgeon at Tiverton Infirmary, and then went to Paris for further medical study; he then paid a visit to America as medical attendant to a patient. On his return, he was appointed House Surgeon at the Swansea Infirmary. In 1882 he commenced private practice in Cheltenham Road, Bristol; and in 1889, on the resignation of Mr. Gardiner, he was appointed Surgeon to H.M. Prison at Horfield, which post he held up to the time of his death. During the whole of his career he was connected with the Bristol Eye Dispensary in Orchard Street, where the loss of his assistance will be much felt.

He did not give up the whole of his time to medical work. When the Horfield School Board was formed in 1893, Dr. Prichard came forward as one of the candidates, and was returned at the head of the poll. He remained a member of the Board, and was most regular at the meetings, occupying the post of Vice-Chairman till the election of 1899, when he did not seek re-election. He was a good botanist and an enthusiastic cyclist, and after the disease from which he died had manifested itself he was often doubtful whether the riding had acted as an exciting cause.

He was a member of the British Medical Association, Fellow of the Royal Meteorological Society, and a member of the Bristol Medico-Chirurgical Society, and was generally to be seen at the meetings, though he seldom brought forward any papers. He was also a member of the Bristol Microscopical Society.

Dr. Prichard was one of the most unassuming of men. A certain shyness he possessed kept him from pushing himself to the front, and sometimes made it difficult for new acquaintances to get on well with him at first, but when once this was overcome he was a most amiable and genial companion. Somehow he always found his way to the heart of children, and he leaves behind him a large circle of friends, young and old, to mourn their loss. He never married.

Acting up to his strong scientific convictions, he expressed a wish that his body should be cremated. This was done at Woking; and a large number of his patients in the neighbourhood in which he lived, the warders from the prison who could be spared from duty, and many members of the medical profession gathered together on February 7th to show their last respect to him, when the coffin containing his ashes was interred in the family vault at Redland Green.

